# From delivery to integration: CRISPR‐mediated large insert gene editing in primary human T cells

**DOI:** 10.1002/cti2.70124

**Published:** 2026-10-18

**Authors:** Ben Buttle, Daniel Verdon, Hilary M Sheppard

**Affiliations:** ^1^ School of Biological Sciences The University of Auckland Auckland New Zealand

**Keywords:** CAR T cells, CRISPR, gene editing, T cell receptors, T cells

## Abstract

Large insert gene editing in *ex vivo*, primary human T cells represents an important approach for engineering next‐generation cellular immunotherapies. While CRISPR‐based editing is well‐established for small genetic modifications, large insert knock‐in remains a technical bottleneck. This review examines current strategies for achieving efficient, precise and safe integration of large genetic elements. We compare delivery methods for CRISPR components, highlighting electroporation of Cas9 ribonucleoproteins as the most efficient but often cytotoxic approach; emerging alternatives such as cell‐penetrating peptides and lipid nanoparticles offer improved cell viability at the expense of editing efficiency. The influence of genomic locus selection, guide RNA design and chromatin context on editing outcomes, as well as the role of homology‐directed repair (HDR) template design is evaluated. Various template formats—including linear double‐ and single‐stranded DNA, plasmids, circular single‐stranded (css) DNA and recombinant adeno‐associated virus (rAAV)—are assessed for their efficiency, toxicity and risk of off‐target integration. Where possible, cross‐study normalisation is used to enable meaningful comparisons. rAAV templates consistently achieve the highest insertion rates but come with concerns regarding cost, size limitations and insertional mutagenesis, whereas non‐viral approaches such as cssDNA templates show promise. Additionally, we review the use of small‐molecule modulators to enhance HDR efficiency, noting trade‐offs between improved insertion rates and potential genomic instability. Overall, successful large insert editing requires balancing of editing efficiency, cell viability and genomic safety considerations. Continued optimisation of delivery systems, template design and repair pathway modulation will be critical for advancing scalable and clinically viable T‐cell therapies.

## Introduction

Large insert T‐cell editing using CRISPR enables the precise integration of large DNA fragments (defined here as > 500 base pairs) into T cells. This approach allows for the engineering of cell‐based immunotherapies with improved functionality and durability. Traditional gene‐editing methods typically favor the generation of small DNA edits but, if this limitation can be overcome, large insert T‐cell editing can be used to engineer sophisticated cell designs for more effective treatments. This review explores the current strategies and emerging challenges in achieving efficient, safe and stable integration of large DNA fragments specifically in *ex vivo* primary human T cells. It also aims to critically assess and compare the efficacy of various CRISPR‐mediated gene editing methods and homology‐directed repair (HDR) template designs.

## T cells as an immunotherapy

T cells are an essential part of the adaptive immune system, capable of targeting and killing cancerous or virally infected cells. Consequently, T cells are being used as a cell‐based immunotherapy to treat some cancers.^1^, ^2^ The first therapy in this class, approved by the US Food and Drug Administration in 2017, is used to treat children with acute lymphoblastic leukaemia.^3^ Since then, other cell‐based therapies have been approved to treat blood cancers, including non‐Hodgkin's lymphoma and multiple myeloma.^4^, ^5^

The success of these therapies relies on the presence of a receptor on the T cell that can recognise and bind to an antigen expressed by the cancerous cell. Binding leads to T‐cell activation and subsequent tumor cell destruction. The receptors can be naturally occurring or ‘replaced’ T‐cell receptors (TCRs, generating TCR‐T cells), such as those expressed by tumor infiltrating lymphocytes (TIL), or engineered such as chimeric antigen receptors (CARs, generating CAR‐T cells).^6^, ^7^, ^8^ The latter contains an antibody‐derived recognition sequence that recognises target antigens fused to a TCR signalling domain.^9^ Several CAR‐T cell products have approvals for use in the clinic for a limited number of indications and targets.^10^ Typically, the DNA encoding the desired receptor is inserted via retroviral or lentiviral delivery but there are several drawbacks with this method.^10^ One is that retroviral insertion results in variable copy numbers of the target gene being expressed under a constitutive promoter. Precise insertion of a CAR downstream of the endogenous TCR promoter is preferable as it allows for dynamic regulation of expression, replicating the dynamic regulation of the TCR after activation and improving anti‐tumor activity.^11^, ^12^, ^13^ Furthermore, in the case of TCR‐T cells, mispairing between endogenous and introduced TCR chains can occur when using a retroviral integration approach, leading to potential incorrect antigen targeting and reduced efficacy.^12^, ^14^ Strategies have been used to avoid this, such as knockout of the endogenous TCR and the engineering of disulphide bonds and/or murinisation of the therapeutic TCR to promote functional pairing. However, receptor engagement is lowered with the latter approach because of the simultaneous expression of the endogenous TCR.^15^, ^16^, ^17^ Finally, the random genomic integration feature of retroviruses comes with a risk of disrupting important functional genes. This ‘insertional mutagenesis’ effect may also, in some cases, lead to the development of cancers.^18^ Indeed, several cases of secondary cancers have been reported following treatment with clinically approved CAR‐T therapies.^19^ Although this has occurred at a very low rate,^20^ it does highlight the potential risk of retrovirus‐based genetic therapy. Moreover, the biological impact of random insertion can be hard to predict and can be patient specific. This is exemplified by a report of altered CAR‐T cell activity because of insertional disruption of the methylcytosine dioxygenase 2 (*TET2*) gene in a patient with a pre‐existing heterozygous loss‐of‐function *TET2* mutation.^21^ Here, disruption of this tumor suppressor and epigenetic regulator enhanced the therapeutic effect.

Additional gene engineering can deliver an optimised T‐cell product with improved anti‐cancer activity. Examples include affinity tuning of the receptor,^22^ regulation of receptor expression^23^ and/or programming the inducible expression of cytokines following activation.^24^ In each case, the limitations of retroviral gene delivery can be addressed using the more precise CRISPR/Cas (clusters of regularly interspaced short palindromic repeats and CRISPR‐associated (Cas) protein) delivery tools.^12^, ^25^, ^26^ However, there are challenges associated with large insert CRISPR‐mediated gene editing, including high levels of toxicity combined with generally low insertion rates. Here, we review the optimisation of methods relating to delivery of the editing machinery, repair template types and the use of HDR enhancers, which may be required to achieve the high cell yields needed for clinical applications.

## The CRISPR/Cas9 editing system

CRISPR was originally discovered as a viral defence system in bacteria.^27^ Following an infection, short DNA sequences from the viral genome are introduced between CRISPR sequences. Upon a repeat infection, the CRISPR sequence and viral sequences are transcribed into a guide RNA (gRNA) and hybridise to a CRISPR‐associated protein (Cas). This protein, guided by the gRNA, can then hybridise to a complementary sequence in the viral genome and cause a DNA break, inactivating the virus. Crucially, no cut is made unless the target site is flanked by a protospacer adjacent motif (PAM) site. As PAM sites are absent in the bacterial CRISPR locus, this mechanism neatly avoids bacterial genome cuts.

Today, this system has been co‐opted for use in mammalian gene editing, allowing for the manipulation of genes for a wide variety of purposes, including cellular immunotherapy. The SpCas9 protein from *Streptococcus pyogenes* has typically been used in mammalian systems.^28^ The on‐target specificity of the Cas nuclease is easily programmed when provided with a user specified guide RNA that has sequence complementarity with a specific genomic DNA sequence.^29^ When this sequence is recognised the Cas9 protein creates a double‐stranded DNA break (DSB) if it is flanked by a PAM site.^27^ The break is repaired through two main pathways that are in competition with each other. The first is non‐homologous end‐joining (NHEJ), which results in imprecise joining of the two DNA ends and typically results in insertion and deletion events (indels), which knock out expression of the gene of interest (GOI).^30^ The second pathway is homology‐directed repair (HDR). HDR requires an HDR template (HDRT) that contains an intended edit flanked by sections of DNA with homology to either side of the DSB.^31^ HDR allows for the insertion of variable lengths of DNA. However, typically gene insertion rates decrease as the size of the insert, and hence the DNA template length, increases (Tables 1, 2, 3).

**Table 1:** Maximum reported insertion rates from each study using linear HDRTs in primary human T cells

Template type	Insertion rate (%)	Insert size (bp)	HA size L/R (bp)	HDRT concentration (nM)^a^	Nuclease	Nuclease delivery	Locus	Insert	Year + Ref.
Hybrid ssDNA^b^	88.75	904	228/368	—	Cas9 RNP	Nucleofection	*IL2RA*	tNGFR	2023^43^
dsDNA	53.7^c^	729	351/303	29.34–58.7	Cas9 RNP	Nucleofection	*CLTA*	GFP	2020
dsDNA^b^	~42	2015	400/399	28.85	Cas9 RNP	Nucleofection	*TRAC*	CD‐19 CAR	2022^71^
dsDNA	40.3	732	306/312	240.45	Cas9 RNP	Nucleofection	*RAB11A*	GFP	2018^25^
dsDNA^d^	~40	756	241/376	118.25	Cas9 RNP	Nucleofection	*IL2RA*	Fluorophore	2022^47^
Hybrid ssDNA nanostructures^b^	~20	2900	300/300	25	Cas9 RNP	Electroporation	*IL2RA*	GFP + EF1a promoter + mCherry	2022^69^
dsDNA	~18	2503	500/500	64.85	Cas9 RNP	Nucleofection	*TRAC*	1G4 TRA + mNeonGreen	2022^44^

^a^ Concentration was determined by converting all masses into molar quantities using the sequence, where available, or the length of the HDRT followed by the division of the molar quantities by the volume of buffer used during HDRT delivery.

^b^ Includes truncated Cas9 target sequence.

^c^ RNP complexation is completed in the presence of PGA.

^d^ DNA chemically modified by interstrand crosslinking.

Recent developments have led to the use of alternative Cas proteins to create distinct types of cuts that require different PAM sequences. For example, AsCas12a from *Acidaminococcus*, which recognises a longer PAM sequence (TTTV), has been used to create staggered DSBs that may promote desirable editing outcomes.^32^ Newer editing modalities such as BASE editors avoid DSBs altogether, relying instead on a Cas9 nickase to deliver a single‐strand nick to the target DNA.^33^, ^34^ Although this leads to a favorable safety profile, base editors are typically limited to single base changes, although they do have the potential for large knock‐ins.^35^ Prime editors, which also rely on a Cas9 nickase, do allow for large inserts, but their use in primary human T cells is currently limited,^36^, ^37^ although rapidly expanding.^38^, ^39^ This review, therefore, focuses on large insert editing mediated by Cas9 as it is the nuclease predominantly used in T cell editing to date.

It is clear from the above that large insert gene editing is a versatile tool for the development of therapeutic T cells. However, consideration must be given to the editing strategy to ensure that optimal editing rates and T‐cell phenotypes are produced following editing. A variety of different methods have been used to generate CRISPR‐mediated gene knock‐ins in primary human T cells. Direct comparison of editing efficiencies between studies is complicated by the different editing protocols used, varied insert sizes, homology arm lengths and diverse target loci. Nevertheless, here we attempt to provide a broad comparison of large insert gene editing strategies to determine the most effective strategies.

## Delivery of the editing machinery

### Electroporation

In primary human T cells, delivery of the nuclease and gRNA has been achieved almost exclusively by electroporation of the Cas nuclease as a ribonucleoprotein (RNP) complex or mRNA.^11^, ^12^, ^25^, ^40^, ^41^, ^42^, ^43^, ^44^, ^45^, ^46^, ^47^, ^48^, ^49^, ^50^, ^51^, ^52^, ^53^, ^54^, ^55^, ^56^ The delivery of nuclease in these forms ensures that its presence in the nucleus is transient, which limits the risks of off‐target cutting or re‐cutting of genomic DNA that has already undergone HDR.^57^, ^58^ Methods for complexation and preparation of a Cas9 RNP prior to electroporation can effect editing outcomes. For example, the addition of negatively charged polymers such as poly(L‐glutamic acid) (PGA) and non‐homologous single‐strand DNA enhancers (ssODNenh) to the RNP can shield excess positive charges on the Cas9 from the bound mRNA, preventing RNP aggregation. This has been shown to improve T‐cell viability and HDR rates following electroporation.^46^ However, deep sequencing in one study revealed a small increase in off‐target cutting when using PGA, therefore, caution should be taken when using this approach.^46^

### Cell penetrating peptides and lipid nanoparticles

Electroporation is efficient but can result in changes in the expression of activation, exhaustion, cell phenotype and toxicity‐related genes, which may result in a negative effect on T‐cell function.^59^, ^60^ Alternative and more gentle strategies of nuclease delivery are being explored which appear to ameliorate these negative effects. Cell‐penetrating peptides (CPPs) are emerging as a promising vector for nuclease delivery. The ‘peptide‐enabled RNP delivery for CRISPR engineering’ (PERC) approach uses a CPP fusion protein of the HIV‐1 TAT sequence with the HA2 peptide from influenza. This likely delivers Cas9 by inducing pinocytosis via the TAT sequence and promoting endosomal escape via the HA2‐derived peptide sequence.^59^ This approach appears to have a minimal effect on the T‐cell transcriptome compared to electroporation. Similarly, delivery of Cas9 within a lipid nanoparticle (LNP) also reduces deleterious transcriptomic changes and may preserve beneficial stem‐memory and central‐memory T‐cell phenotypes.^60^, ^61^ However, neither approach has achieved the same level of DNA cleavage compared to electroporation. For example, CRISPR Cas9 editing via electroporation induced gene knockout in approximately 30% more cells compared to PERC at the same locus, but higher levels of toxicity meant that it resulted in a significantly lower yield of edited cells.^59^

One major limitation of the CPP system is that the addition of a dsDNA HDRT required to achieve HDR significantly reduces cutting, probably because of competition between the Cas9 RNP and HDRT for CPP complexation.^59^ This issue can be circumvented by using a recombinant adeno‐associated viral vector (rAAV) HDRT, which can enter the cell nucleus independent of the CPP.^59^ HDR using LNPs in T cells has also only been achieved using rAAV vector‐mediated delivery of the HDRT, and further research is required to determine whether this is necessary for high HDR rates.^60^

## Locus‐dependent effects

### Locus dependent editing efficiency

The target locus for DNA insertion plays a role in editing efficiency as the capacity of Cas9 to bind DNA and generate a DSB is both sequence‐ and locus‐dependent. GC content, chromatin state and target gene function all contribute to cutting and HDR rates. Studies have shown that a GC content of 40–60%, with lower than 56% GC content in the PAM‐proximal region (within 1–12 nucleotides of the PAM site) and higher than 50% GC content in the PAM‐distal region is optimal for gRNA cutting in mammalian cells.^62^, ^63^, ^64^

The genomic sites that can be effectively targeted by a gRNA are also limited by the DNA sequence at a specific locus. For CRISPR Cas systems, the target site must be flanked by a PAM sequence (*e.g*. NGG for Cas9, TTTV for AsCas12a), which may impact optimal gRNA design. In addition, cutting efficiency is demonstrably influenced by locus‐dependent factors, as gRNAs targeting different loci with the same sequence exhibit varying cleavage efficiencies.^65^ This is likely because of differences in DNA availability driven by factors such as chromatin state and DNA methylation.^66^ Therefore, choice of target locus for large insert integration is important and may also be exploited to ensure high editing rates. For example, targeting an essential gene such as *GADPH* with an HDRT containing a restorative sequence followed by a GOI results in the death of cells that have been cleaved but not undergone HDR, thus selecting for cells that have undergone HDR.^53^ While this strategy results in cell populations with a large proportion containing the intended edit (> 90%), it does mean that the inserted gene will be under the control of the gene promoter of the target locus, which may not be ideal for all therapeutic purposes.

### Locus dependent effects on functionality

In addition to affecting editing rates, the selected target site for transgene integration can impact other factors such as safety. One advantage of insertion into the *TRAC* locus is that it knocks out native TCR expression as referred to above. This increases safety by reducing the potential for off‐target effects mediated by mispairing between endogenous and introduced TCR chains.^12^, ^14^ It is also considered a safe harbour locus for transgene integration as it is a well‐characterised site that supports transgene expression without affecting genomic integrity or cellular activity. Other safe harbour sites include AAVS1, ROSA26 and CLYBL where targeted transgene insertion should avoid insertional mutagenesis.^67^

A strategic choice of transgene insertion site can also positively impact T‐cell phenotype. This is an important consideration as early memory phenotypes exhibit superior long‐term anti‐tumor persistence compared to more differentiated effector cells.^68^ Here again, transgene insertion at the TRAC site appears superior to other sites as endogenous dynamic regulation of the receptor delays effector T‐cell differentiation and exhaustion and improves anti‐tumor activity.^11^ Furthermore, the previously noted random insertion of a CAR‐T into the *TET2* locus promoted a central memory phenotype with an effective anti‐tumor response, clearly highlighting the impact of locus dependent effects on cell phenotype and activity.^21^

## Template design for homology‐directed repair

Optimised template design is essential for successful large insert gene editing. The genetic composition, intended function and size of the insert all play important roles in determining editing and functional efficiency. Two main design strategies have been employed to date in large insert T‐cell editing (Figure 1). The first is to introduce new genetic components to an endogenously expressed gene, such as in TCR redirection (Figure 1a).^12^, ^25^, ^40^, ^44^ This approach allows for functional alterations to the protein with a reduced insert size. The second approach introduces an entirely new gene into an existing locus. This knocks out the endogenous gene and puts the insert under the control of the endogenous promoter, such as in CAR‐T cell generation (Figure 1b).^11^, ^48^, ^55^ This approach can also be used with a template that restores the complete endogenous gene along with the intended insert, allowing for the expression of both proteins under the original promoter.^53^ However, this increases HDRT size, which may have adverse effects on HDR rates. A third, less common, approach is to insert a GOI with a promoter, removing the need for an endogenous promoter.^69^, ^70^

**Figure 1:**
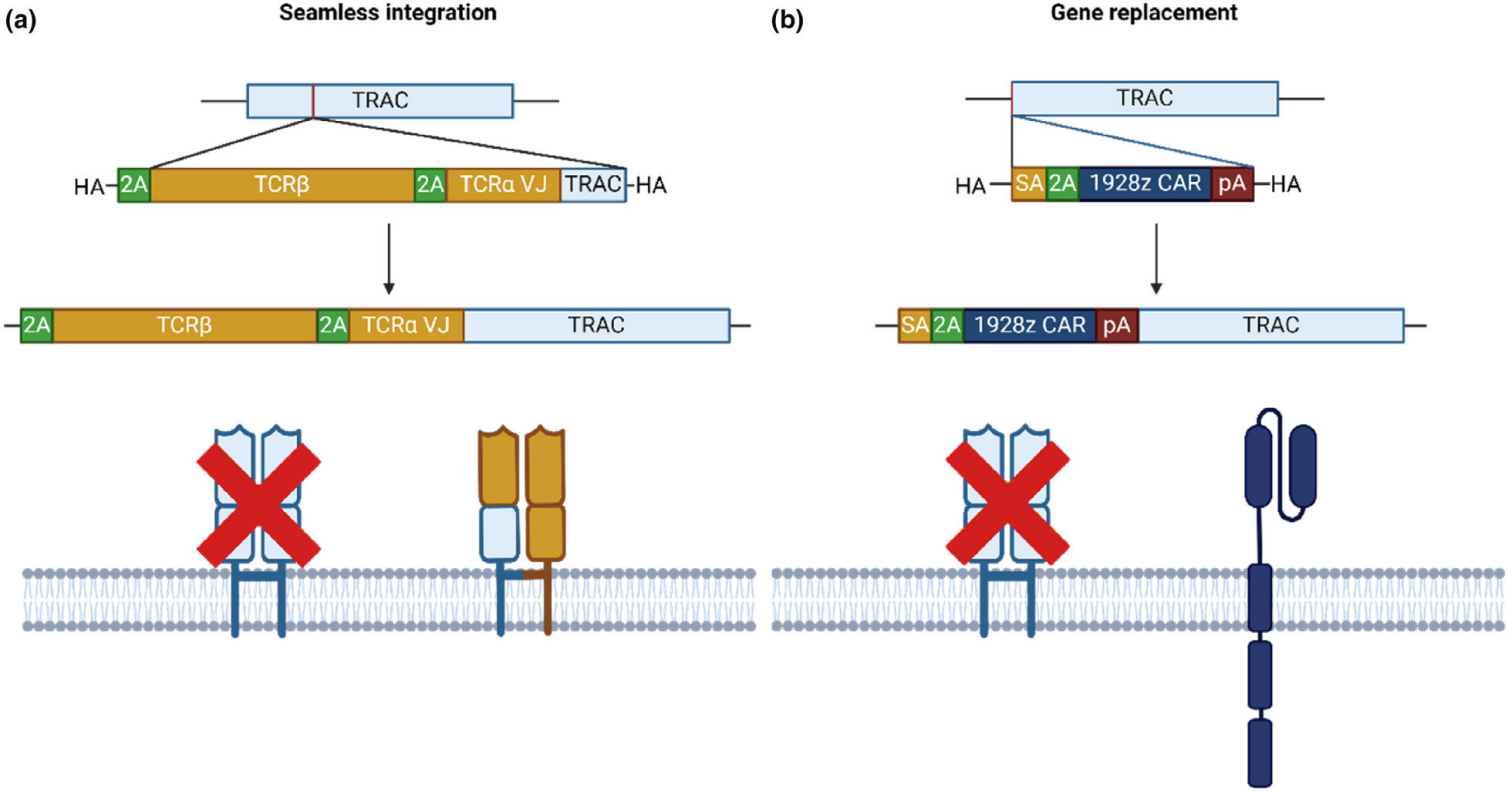
Large insert HDRT design strategies for therapeutic T‐cell editing. **(a)** Insertion of complete TCRβ and TCRα into the TRAC locus, creating a fusion protein that maintains components of the original TCRα gene under the control of the endogenous TCRα promoter. **(b)** Insertion of a 1928z car into the TRAC locus, knocking out the endogenous gene and putting the CAR under the TCRα promoter. 2A symbols represent 2A self‐cleaving peptides, SA represents splice acceptor sites, pA represents a polyA sequence, and red line indicates a DSB site.

### Template structure

The structural design of HDRTs, such as the choice of linear, circular, double‐stranded, single‐stranded DNA or rAAV templates, has important implications on HDR rates, toxicity, and off‐target insertion editing in primary human T cells (Tables 1, 2, 3).

#### Linear dsDNA

##### Template Length

Long, linear double‐stranded DNA (dsDNA) HDRTs are commonly used in T‐cell editing methods (Table 1). Their length allows them to encompass multiple protein‐coding genes. They are simple to generate and purify, and are relatively inexpensive, making them an attractive template type for cellular immunotherapy protocols. They are typically plasmid‐derived or PCR‐amplified and, like all HDRTs, they contain the gene/s of insert flanked by homology arms.

Delivery of linear dsDNA HDRTs to primary human T cells is typically achieved through simultaneous electroporation of the dsDNA with complexed CRISPR/Cas9 RNPs.^12^, ^25^, ^40^, ^43^, ^46^, ^47^, ^52^ In the absence of modifications to this protocol, HDR rates appear to be driven by the amount of template supplied during editing with high insertion rates driven by high concentrations of the HDRT. However, the maximum concentration of template that can be supplied to the cells is dependent on HDRT length because as the length of a dsDNA increases, so does its toxicity.^25^, ^72^ This is because intracellular DNA sensors such as cyclic guanosine monophosphate‐adenosine monophosphate synthase (cGAS) bind unprotected cytoplasmic DNA in a length‐dependent manner, leading to cell death via Stimulator of Interferon Genes (STING) signalling.^72^

It is difficult to make meaningful comparisons between studies because of multiple variables. However, in an attempt to make useful comparisons between published studies, we calculated the concentrations of HDRTs used as nanomolar amounts (nM). This metric considers both the concentration of the template used and its length. Table 1 lists just the highest editing rates achieved within assorted studies using linear ss and dsDNA HDRTs. Our analysis highlights that shorter templates can be tolerated at higher concentrations. For example, insertion of an eGFP tag into the RAB11A locus was achieved in more than 40% of T cells using a high concentration, 240 nm, of a 1350‐bp‐long dsDNA HDRT.^25^ However, to insert a larger TRA plus NeonGreen element, the highest concentration of a > 2200‐bp‐long dsDNA HDRT that resulted in approximately 24% of edited cells and the highest overall edited cell yield was less than one‐third of this at ~65 nm.^44^

##### Chemical modifications

Modifications to dsDNA HDRT physical structure can increase HDR rates. For example, interstrand crosslinking using psoralen or cisplatin resulted in an up to fivefold increase in HDR rates when using a ~1400‐bp‐long HDRT in primary human T cells.^47^ This may be because interstrand crosslinks in the HDRT act as a canonical substrate for the Fanconi anaemia pathway which targets them for repair.^73^ However, this pathway is now also recognised as playing an important role in HDR following CRISPR/Cas9 cutting when using both dsDNA and ssDNA HDRTs.^74^

Another explored modification is the inclusion of truncated Cas9 target sequences (tCTS) at the ends of a HDRT. Here, a sequence containing a PAM site and 16‐bp complementary to the gRNA allows Cas9 to bind, but not cut the HDR template, which facilitates shuttling into the nucleus.^43^, ^46^, ^69^ This approach improved HDR efficiency approximately twofold compared to templates without tCTS at several loci in both CD4^+^ and CD8^+^ T cells.^43^, ^46^, ^71^ Orientation of the tCTS is important, as only sequences containing the PAM facing towards the centre of the insert sequence improved HDR efficiency.^46^ Including tCTS sequences in a ~1400‐bp‐long HDRT improved HDR rates more than eightfold in bulk T cells and more than fourfold in CD8^+^ T cells, in both cases achieving efficient insertion in more than 50% of cells.

Collectively, these studies demonstrate that a linear dsDNA HDRT can be effective when designed carefully. A limitation however, is that in addition to activating the cGAS pathway,^72^ the presence of DNA ends can be recognised by Ataxia‐telangiectasia mutated (ATM) protein, which further activates nuclear DNA sensing mechanisms that can initiate apoptosis.^75^ Linear dsDNA also has a propensity to insert randomly into off‐target locations as it is an ideal substrate for error‐prone DNA repair pathways and degrades less rapidly than single‐stranded DNA. This means that it is more available to incorporate during regular DNA repair, which is not ideal for gene editing and therapeutic purposes.^25^

#### Linear ssDNA

Linear ssDNA (lssDNA) is frequently used as a HDRT in T cells. ssDNA is a less potent activator of intracellular DNA sensors, reducing its toxicity compared to dsDNA.^76^ Additionally, ssDNA is integrated into genomic DNA through a different mechanism compared to dsDNA.^77^ This appears to be more reliant on the Fanconi‐anaemia pathway compared to dsDNA, but the exact mechanism is not yet well‐understood.^74^

When supplied at equivalent concentrations in human cells, lssDNA leads to lower levels of off‐target integration compared to dsDNA but also produces lower on‐target integration efficiencies.^25^, ^43^, ^78^ However, as this is accompanied by a higher viability a higher concentration of template can be used, which improves the overall yield of edited cells.^43^ Roth *et al*.^24^ observed that electroporation of 6 μg of a 1.3‐kb ssDNA HDRT resulted in an approximately 75% viability compared to the approximately 1% viability when using 6 μg dsDNA HDRT. This reduced toxicity of linear ssDNA templates compared to dsDNA templates is consistent across studies using dsDNA and highlights the advantage of using a lssDNA template to maintain a viable population of edited T cells.

##### Linear ssDNA production

The production of lssDNA HDRTs can be achieved using a variety of methods. One is through amplification of linear dsDNA using a single biotinylated primer. The resulting biotinylated amplicon is bound to streptavidin‐coated beads, dissociated with a high salt buffer, allowing for the collection of the non‐biotinylated strand.^43^ Other methods include amplification of dsDNA with a single phosphorylated primer, followed by enzymatic digestion of the phosphorylated strand or reverse transcription of an RNA intermediate, followed by hydrolysis of the RNA strand.^25^ Although these methods may allow for relatively low‐cost production of ssDNA, caution and careful validation are necessary, as contamination with dsDNA in the final product is possible and may skew results.^79^

##### Addition of truncated Cas9 target sequences

Truncated Cas9 target sequences, as discussed above for dsDNA, have also been used to improve HDR rates using ssDNA templates. This requires the generation of hybrid dsDNA/ssDNA constructs where the bulk of the HDRT is single‐stranded, and the end tCTS sequences are double‐stranded to allow Cas9 to bind.^43^, ^69^ These double‐stranded sequences facilitate similar HDR rates when generated either as hairpins or with hybridised complementary oligos.^43^ This template type consistently led to an increase in HDR rates compared to standard lssDNA in two studies, although the extent of the increase appears to be dependent on the locus, HDRT length and structural conformation of the ssDNA molecule (Table 1).^43^, ^69^ The hybrid template also appeared to significantly outcompete dsDNA templates containing identical sequences and a tCTS in both HDR rates and viability.^43^ The higher editing rates are likely because of a combination of an increase in HDR through ssDNA HDR mechanisms, proximity of the HDRT to DSB sites driven by the tCTS, and an increase in the amount of overall HDRT that can be supplied.

#### Circular DNA

##### Circular double stranded DNA

Plasmid DNA is a commonly used HDRT type for large insert gene editing because of the ease of production and purification compared to other template types. In T‐cells, plasmid DNA is typically delivered by electroporation simultaneously with a Cas9 RNP. However, their use in primary human T cells is limited (Table 2) probably because of the toxicity associated with the plasmid size^84^ as discussed above. Plasmid HDRTs contain kilobase‐sized regions of extraneous backbone DNA, which limits the amount of template that can be delivered to T‐cells before there is a significant reduction in viability. Additionally, plasmid size affects DNA entry into the cell during electroporation. Accordingly, various strategies have been developed to circumvent these issues.

**Table 2:** Maximum reported insertion rates for circular HDRT vector types from each study using circular HDRTs in primary human T cells

Template type	Insertion rate	Insert size (bp)	HA size L/R (bp)	HDRT concentration (nm)^a^	Nuclease	Nuclease delivery	Locus	Insert	Ref.
Nanoplasmid	46.3–57.9%	1789	500/500	95.05	Cas9 RNP	Electroporation	*TRAC*	CD19‐CAR	2022^44^
cssDNA	56.3%^b^	1764	302/308	113.00	Cas9 RNP	Nucleofection	*TRAC*	CD19/CD22 dual CAR	2024^49^
Nanoplasmid^b^, ^c^	~45%	3226	396/331	35.97	Cas9 RNP	Electroporation	*TRAC*	tEGFR + NY‐ESO‐1 TCR	2025^80^
pRL‐142 plasmid	~40%	2471	1021/1048	44.96	Cas9 RNP	Nucleofection	*TRAC (intron)*	GFP + CAR	2025^81^
pUC57 plasmid	~40%^b^	1263	500/500	40.72	Cas9 RNP	Electroporation	*TRAC*	NY‐ESO‐1 TCR + mNeonGreen	2022^44^
cssDNA	34%	2490	302/308	~90	Mod Cas9 mRNA^e^	Nucleofection	*TRAC*	CD19/CD22 dual CAR	2025^82^
cssDNA	33.4%	1689	300/300	144.3	TALEN mRNA	Electroporation	*B2M*	Cell surface reporter	2025^83^
pUC57 plasmid^c^	13.2%	3700^d^	–	63.32	Cas9 RNP	Electroporation	*TRAC*	CD19‐CAR	2021^50^
pMini plasmid^c^	16.8%	3700^d^	–	76.58	Cas9 RNP	Electroporation	*TRAC*	CD19‐CAR	2021^50^
Plasmid	~3%	2000	300/300	104.75	Cas9 RNP	Nucleofection	*RAB11A*	GFP	2024^49^

^a^ Concentration was determined by converting all masses into molar quantities using the sequence, where available, or the length of the HDRT followed by the division of the molar quantities by the volume of buffer used during HDRT delivery.

^b^ Rates were achieved using an HDR enhancer.

^c^ Includes CTS.

^d^ Length includes HA.

^e^ Cas9 fused to a single‐stranded DNA binding peptide motif.

The inclusion of a Cas9‐cleavage sequence (CCS) flanking the homology arms has been used to effectively excise and linearise a HDRT from plasmid DNA without the need for PCR and further purification steps.^50^, ^85^, ^86^ This process may also facilitate guiding of the cleaved HDRT to the nucleus through Watson‐Crick‐Franklin interactions, improving HDR rates via mechanisms similar to those discussed above in relation to tCTS.^43^, ^46^, ^50^ Indeed, the inclusion of two CCS signals in a plasmid was reported to increase HDR rates up to 10‐fold.^50^ However, despite this increase, HDR rates using these plasmid templates remain relatively low compared to other template types, with a reported maximum HDR rate of 13% on average across multiple donors.^50^

Plasmids with size‐reduced backbones have been developed for use as HDRTs in primary human T cells (Table 2), including nanoplasmids and pMini.^44^, ^50^ pMini is a pUC57 plasmid (~2.7 kb) with the lacZ reporter gene removed to create a ~1.6‐kb backbone.^50^, ^80^ However, despite its reduced size, it has not been demonstrated to improve viability or HDR rates compared to HDRTs with a complete pUC57 backbone.^50^ Nanoplasmids are even smaller, as the antibiotic resistance selection marker is replaced by an RNA‐OUT selection marker, reducing the backbone to fewer than 500 bp.^87^ In a direct comparison, when used as an HDRT at optimised concentrations, nanoplasmids led to improved viability and overall yield of edited cells compared to both a pUC57 plasmid and a linear dsDNA HDRT containing the same 1263‐bp‐long insert (120.7 nm nanoplasmid yielded up to 46.6% insertion; 32.6 nm plasmid yielded up to 36.2% insertion; 32.4 nm linear DNA yielded up to 20% insertion).^44^ Both the nanoplasmid and pUC57 plasmid HDRTs delivered improved insertion rates compared to dsDNA, possibly because circular DNA is more stable intracellularly than linear dsDNA.^88^ The reduced toxicity is likely a factor of both reduced DNA length and a lack of DNA ends leading to reduced activation of DNA sensing pathways.^72^

##### Circular single‐stranded DNA

Recently, circular single‐stranded DNA (cssDNA) has been developed for use as HDRTs. These templates combine the benefits of ssDNA with circular HDRTs. We are aware of three studies to date that have demonstrated that the use of cssDNA HDRTs results in higher HDR rates compared to linear ssDNA and circular dsDNA HDRTs in human cell lines,^79^ haematopoietic stem cells,^83^ and primary human T cells.^49^, ^83^ One further recent study in primary human T‐cells demonstrated enhanced HDR rates using cssDNA templates when combined with a Cas9 molecule engineered to bind to a single‐stranded DNA binding protein motif.^82^ This is proposed to recruit the cssDNA template to the HDR site and increase its localised concentration and hence efficient integration. However, use of this HDRT type in primary human T cells is limited and further data is required to determine donor‐to‐donor variability, off‐target integration rates and to directly compare the efficacy of this template to other HDRT types.

#### AAV

AAV‐based HDRTs are an attractive option for large insert gene editing as they can insert large gene cargos at a high rate compared to non‐viral approaches. Recombinant AAV HDRTs have been used in combination with a variety of nucleases, including CRISPR, TALENs and megaTAL, and in combination with a variety of delivery mechanisms, including electroporation, LNPs and CPPs.^41^, ^54^, ^55^, ^56^, ^59^, ^60^ This versatility is because rAAV HDRTs are delivered directly to the nucleus of the cell through the innate intracellular trafficking processes of the AAV, which may increase local HDRT concentration and contribute to higher HDR rates.^91^

The structure of AAV HDRTs within the vector backbone is similar to linear ssDNA HDRTs. However, the 5′ and 3′ ends contain a short inverted terminal repeat (ITR) sequence that forms two hairpin loops which serve as the origin of viral genome replication and which improve stability of the template.^92^ rAAV HDRTs generally show optimal editing with moderate homology arm (HA) lengths (~500–700 bp). While efficient HDR has been reported using longer arms ranging from 500 to 1300 bp, they do not consistently improve outcomes, and high efficiencies (up to ~90%) have been achieved using ~550–600 bp arms, even for kilobase‐scale inserts.^53^, ^54^, ^56^ As both longer and shorter arms have improved editing in specific contexts, this suggests that locus, cell type, and insert size influence results, while asymmetric arms offer no clear benefit.^41^, ^51^, ^93^ T cells are also more tolerant to rAAV template length compared to naked DNA templates (Tables 1, 2, 3). This is because HDRTs within rAAVs are protected inside the virus capsid until they reach the nucleus, where uncoating occurs, and thus they avoid activation of the DNA sensing pathways.^94^, ^95^

**Table 3:** Maximum reported insertion rates in large insert rAAV HDRT gene editing studies

Insertion rate (%)	Insert size (bp)	HA size L/R (bp)	Nuclease	Nuclease delivery	Locus	Insert	Ref.
95.3	–	–	TALEN mRNA	Electroporation	*B2M*	HLAE	2022^55^
92	1126	544/616	AsCas12a RNP	Electroporation	*GADPH*	GFP	2024^53^
86.2	1500–2000	–	AsCas12a RNP	Electroporation	*TRAC*	1928z CAR	2023^59^
82	1800	1300/1300	megaTAL mRNA	Electroporation	*CCR5*	GFP	2015^54^
76.54	2100	~700/700	Cas9 RNP	Electroporation	*Thy1*	GFP	2026^89^
75.1	1500–2000	–	AsCas12a RNP	PERC CPP	*TRAC*	1928z CAR	2023^59^
60–70	–	–	Cas9 RNP	Electroporation	*TRAC*	Wt‐1 TCR	2022^45^
46.7	2276	958/1000	TALEN mRNA	Electroporation	*CD40LG*	GFP	2016^56^
46	2500	400/400	Cas9 RNP	Electroporation	*CCR5*	GFP	2017^90^
45.6%	–	–	Cas9 mRNA	Electroporation	*TRAC*	1928z CAR	2017^11^
> 45	–	476/1428	ZFN mRNA	Electroporation	*CCR5*	GFP	2016^51^
~40	–	–	Cas9 mRNA	Electroporation	*CD40LG*	EGFR	2023^60^
~40	2200–2700	600/600	megaTAL mRNA	Electroporation	*TRAC*	CD19‐CAR	2017^41^
~25	–	–	Cas9 RNP	LNP	*CD40LG*	EGFR	2023^60^
14.344	~2466	700/700	Cas9 RNP	Electroporation	*CX3CR1*	TCF1	2026^89^

NB – MOI not reported because of variability in quantification between studies. –, Data not available.

AAV HDRTs have a limited total size capacity of approximately 4.7 kb. This may be problematic as increasingly complex and larger gene cargoes are being designed. Bak & Porteous^90^ addressed this with multiplexed editing, where two rAAV donor templates were used simultaneously, the first containing homology arms to genomic DNA and the second containing arms with homology to the first donor template. The first HDRT reconstituted the original gRNA target site, allowing the CRISPR machinery to re‐cut the DNA following HDR to allow for a subsequent HDR event using the second rAAV HDRT. This strategy facilitated the insertion of a 6.5‐kb gene cassette containing a full‐length EGFR gene with regulatory elements in ~10% of primary human T cells. The simultaneous addition of both rAAV HDRTs immediately following electroporation of the Cas9 RNP may limit the HDR capacity because of degradation of the RNP before a second round of HDR can occur. However, sequential editing is not recommended because of the potential for reduced tolerance of repeated genetic manipulations.

rAAV vectors are generally considered non‐integrative, which contributes to their utility as HDR templates. However, they can integrate through non‐homologous mechanisms. Although the presence of homology arms significantly increases targeted integration rates, rAAV HDRTs have been shown to integrate into a target site even in the absence of homology arms at a rate of up to 6%.^54^ This integration is driven by the ITR sequences and can also occur at any break sites.^96^, ^97^ Such non‐homologous integration may not be an issue for expression of the inserted gene in cases where the rAAV template contains both the gene and promoter sequence. However, it would be problematic where seamless integration of a promoterless gene is required, such as with TCR replacement.^98^ Furthermore, the observed prolonged persistence of the template as episomal DNA elevates the risk of off‐target insertion by extending its exposure time to months in the nucleus and, in fact, some insertional mutagenesis has been linked to rAAV vector integration.^99^, ^100^, ^101^ Recently, a young boy developed a neuroepithelial tumor following a gene therapy using AAV‐9; this has been linked to insertion of AAV DNA into the pleomorphic adenoma gene 1 (PLAG1).^102^ Although such cases are rare, this highlights the need for caution when using AAV templates. These insertion events may go undetected as they may occur beyond the typical window for monitoring off‐target effects. Therefore, careful monitoring of off‐target and on‐target integration is necessary to minimise risks to patients receiving T‐cell therapies generated using rAAV HDRTs.

#### Homology arm length

Homology arm length does not seem to significantly affect edited cell yields. Experiments in mammalian cell lines indicate that homology arms from approximately 400–800 bp result in similar HDR rates.^103^, ^104^ One study, using linear dsDNA, has shown that CAR^+^ T cell yield was maximised as homology arm length increased up to 800 bp at the *AAVS1, PD1, TRAC and B2M loci*.^105^ As the homology arm length increased, there was an overall decrease in viability but increased HDR rates compensated for this.^105^ This effect was lost with 1600‐bp homology arms, with no further increase in HDR and significantly higher toxicity. In contrast, optimisation in the TRAC locus using circular dsDNA donors showed improvements in HDR rates as the homology arms increased to 500 bp with no further improvements to HDR or reductions to viability as the homology arm length increased beyond that.^44^ As far as we are aware, direct comparisons like these have only been reported twice^41^, ^98^ using limited gene inserts and loci, so further optimisation experiments are warranted.

## Small‐molecule inhibitors

Small‐molecule inhibitors of proteins involved in different DNA repair pathways have been used to increase HDR rates in T cells. The processes of NHEJ and HDR compete when repairing DNA breaks.^106^ Therefore, inhibition of proteins that initially commit DSBs to the NHEJ pathway can increase HDR rates.^74^, ^107^ The DNA protein kinase catalytic subunit (DNA‐PKcs) is an essential component of the DNA protein kinase (DNA‐PK) complex, which forms early in the NHEJ repair pathway.^108^ Multiple small molecule inhibitors targeting this protein have been developed, including M3814 and NU7441.^109^, ^110^ These inhibitors have been reported to enhance HDR rates in primary human T cells by approximately 49% and 43% respectively when using short templates.^43^ When using a long linear template, HDR was increased by ~15% when using NU7441, and with a plasmid‐based template, HDR was increased by ~23% when using M3814 and by ~16% when using another DNA‐PK inhibitor PI‐103.^43^, ^53^, ^80^ A similar increase was seen when using M3814 with a large insert cssDNA template.^49^ Simultaneous use of these compounds does not further increase HDR rates, likely because of the redundancy of the molecular pathway being targeted.^43^ Although DNA‐PK inhibitors result in a significant increase in HDR, the DNA‐PK inhibitor AZD7648 has now also been implicated in the generation of kilobase to megabase‐sized deletions, chromosome arm loss, and translocations following CRISPR editing, prompting a need for caution with their use.^111^

NHEJ and HDR are the two pathways predominantly cited when discussing DNA repair following CRISPR‐based gene editing. However, other repair pathways can be involved, including the theta‐mediated end joining (TMEJ) pathway, which relies on DNA polymerase θ (Pol θ). This pathway uses regions of microhomology on the DNA break ends (≥1 bp) to anneal the ends together. Dual inhibition of DNA‐PK and Pol θ has been shown to have an additive effect on HDR rates. For example, HDR mediated‐insertion of ~1‐kb template encoding GFP into T‐cells increased 1.4‐fold with dual inhibition compared to DNA‐PK inhibition alone.^107^ The same study also demonstrated reduced off‐target effects using this method in 293 T cells, likely driven by reduced availability of non‐HDR DNA repair pathways. The large genomic deletions, chromosome arm loss and translocations associated with the use of AZD7648^111^ may also be mediated by non‐HDR DNA repair pathways, and so use of a pol theta inhibitor may ameliorate this deleterious effect.^107^

Another pathway that can be targeted to improve HDR rates involves DNA condensation. Histone deacetylases (HDACs) remove acetyl groups from histones, typically resulting in the condensation of DNA. HDAC inhibition (HDACi) can be used to prevent this process, resulting in a more open DNA configuration.^112^ The HDACi trichostatin A (TSA) has been observed to improve CRISPR/Cas9 mediated HDR rates potentially because a more open chromatin state facilitates the binding of proteins involved in HDR.^113^ Additionally, treatment with TSA maintains high levels of acetylation on Ku70/80 which reduces recruitment of NHEJ proteins and facilitates the recruitment of HDR proteins to the DSB site.^114^ TSA has been used in primary human T‐cells to increase the HDR rate by 16% compared to baseline editing rates.^43^ However, use of TSA as an HDR enhancer may be limited as it is toxic at low concentrations because of the untargeted mechanism of action. In addition, the temporary alterations to global chromatin structure may negatively impact T‐cell phenotypes.

Cell cycle is another factor that can affect HDR rates. HDR is cell cycle‐dependent because of the regulation of HDR activity by cyclin‐dependent kinases (CDKs) and the expression of cell‐cycle‐dependent factors.^115^ It is primarily favored in the S/G2 phases of the cell cycle and so inhibition of cell cycle progression can be used to increase HDR rates. For example, transient inhibition of the cell division cycle 7‐related protein kinase (CDC7) using the small inhibitor XL413 acts to synchronise cells in S phase, promoting HDR rates.^52^ Its use in primary human T cells has been shown to significantly increase HDR rates by ~50% above baseline rates.^43^ Despite this, the total number of cells post editing was reduced because of the inhibition of DNA replication that accompanies the inhibition of cell cycle progression.^43^ These effects may be present in other studies, but are not apparent as editing outcomes are often measured as a percentage of cells rather than the total number of cells produced.^52^

Combining small‐molecule inhibitors with distinct mechanisms of action can enhance HDR rates more effectively than a single inhibitor alone.^43^ A combination of M3814, TSA and XL413 has been used together to increase HDR rates at a variety of different loci of potential clinical relevance in T cells.^43^ The increase observed when using these small molecule cocktails was variable, with a minimum of a ~1.1‐fold change at the *CD7* locus and a maximum of a ~9.3‐fold increase in HDR at the *PI3KCD* locus.^43^ The maximum amount of editing achieved using a combination of these inhibitors was ~90% up from a baseline of ~51% HDR using a single‐stranded HDRT with CTSs. Illumina sequencing of PCR amplicons was used to determine on‐ and off‐target events for the integration of a CAR into the TRAC locus; this revealed no off‐target integration, 37% perfect HDR, and 7% imperfect HDR. This level of analysis was only performed for this single locus, and we note that amplicon sequencing may miss the detection of larger deletions, as amplicon primer sites may be located within a deletion. Therefore, further analysis is required to ensure the safety of this type of approach.^116^

## Conclusion

Large insert gene editing in primary human T cells is an exciting prospect for the development of safer and more effective cellular immunotherapies. It can be combined with gene knockout and fine‐tuning of inhibitory molecules to generate T cells with improved cancer‐killing ability.^23^, ^117^ Determining the optimal strategy for engineering edits using CRISPR/Cas9 remains challenging; the balance between editing efficiency and cell viability is yet to be perfected and it is currently unclear how important gene expression levels are, as well as whether a viral or completely non‐viral system is more appropriate. If the potential immunogenicity or size restraints of using viral vectors for HDRT delivery are not an issue, rAAV HDRTs are well‐studied and provide the highest known editing rates but come with limitations including the complexities of episomal persistence, manufacturing to GMP grade and high cost. Currently, the best strategy for non‐viral genome editing in T cells appears to be co‐electroporation of Cas9 RNP with a cssDNA HDRT. However, data are limited and this requires further research. The inclusion of a tCTS has shown promise in a variety of HDRT types. It would, therefore, be interesting to determine whether the inclusion of a tCTS on already high‐performing template types such as cssDNA would further improve HDR rates. Ultimately, refining these approaches will be crucial for enhancing the efficiency, affordability and scalability of engineered T‐cell therapies, paving the way for safer and more effective cancer immunotherapies. Future research could focus on systematically comparing HDR strategies, optimising template design, and further elucidating the mechanisms governing successful gene integration to fully realise the potential of gene‐edited T cells in clinical applications. In addition, in this fast‐moving field alternative editing modalities which rely on prime editing are emerging including PASTE,^118^ PASSIGE,^119^ PRIME‐in^38^ and QUAD‐PE.^39^ These systems avoid the generation of dsDNA breaks and thus reduce the risk of genomic instability.^33^ They have the potential to supersede CRISPR/Cas9 mediated editing as they are increasingly optimised to yield high levels of precisely edited cells.

## Author contributions

**Ben Buttle:** Writing – original draft; writing – review and editing; data curation; conceptualization; methodology; visualization. **Daniel Verdon:** Writing – review and editing. **Hilary M Sheppard:** Conceptualization; project administration; resources; supervision; writing – review and editing.

## Conflict of interest

The authors declare no conflict of interest.
